# Large-Range Polymer Optical-Fiber Strain-Gauge Sensor for Elastic Tendons in Wearable Assistive Robots

**DOI:** 10.3390/ma12091443

**Published:** 2019-05-03

**Authors:** Jonathan Casas, Arnaldo Leal-Junior, Camilo R. Díaz, Anselmo Frizera, Marcela Múnera, Carlos A. Cifuentes

**Affiliations:** 1 Biomedical Engineering Department, Colombian School of Engineering Julio Garavito, Bogotá 111166, Colombia; marcela.munera@escuelaing.edu.co (M.M.); carlos.cifuentes@escuelaing.edu.co (C.A.C.); 2 Graduate Program of Electrical Engineering, Federal University of Espirito Santo, Vitoria 29075-910, Brazil; leal-junior.arnaldo@ieee.org (A.L.-J.); c.rodriguez.2016@ieee.org (C.R.D.); frizera@ieee.org (A.F.)

**Keywords:** physical human–robot interaction, soft robotics, optical-fiber strain gauge

## Abstract

This paper presents the development and validation of a polymer optical-fiber strain-gauge sensor based on the light-coupling principle to measure axial deformation of elastic tendons incorporated in soft actuators for wearable assistive robots. An analytical model was proposed and further validated with experiment tests, showing correlation with a coefficient of R = 0.998 between experiment and theoretical data, and reaching a maximum axial displacement range of 15 mm and no significant hysteresis. Furthermore, experiment tests were carried out attaching the validated sensor to the elastic tendon. Results of three experiment tests show the sensor’s capability to measure the tendon’s response under tensile axial stress, finding 20.45% of hysteresis in the material’s response between the stretching and recovery phase. Based on these results, there is evidence of the potential that the fiber-optical strain sensor presents for future applications in the characterization of such tendons and identification of dynamic models that allow the understanding of the material’s response to the development of more efficient interaction-control strategies.

## 1. Introduction

In recent years, wearable robotics has gained significant attention [1]. This field concentrates on the development of devices that are designed and shaped in functions of the human body and the degrees of freedom of specific joints [2]. In the context of rehabilitation and assistance, wearable robots are mainly focused on the fabrication of exoskeletons (e.g., upper- and lower-limb exoskeletons) and orthoses [3]. The aim of these devices is to provide support to users in different ranges of movement by stabilizing their limbs and helping to restore or reinforce weak functions [4,5]. Among multiple goals of robotic orthoses, they are designed to assist weak muscles, provide adequate stiffness in specific joints, and limit movement range to avoid injuries [4]. Considering that assistive wearable robots, such as exoskeletons and orthoses, are meant to be coupled with human joints, they are designed to adapt physically and cognitively to the users, being able to support them to effectively complete their therapeutic tasks [2]. Therefore, bioinspired designs have been explored, as they allow wearable devices to better interact with the user in terms of lower muscular activation and better kinematic compatibility [5]. However, a current limitation in design is the selection of actuators, as they need to be compliant and back-drivable to meet the needs of devices and their users [5], which, at the end, influences the performance and acceptance of the robotic platform [2,6].

Compliant robotics considers the development of soft robots or actuators with mechanically (or passively) compliant joints with variable joint stiffness [7]. From this approach, series-elastic actuators have emerged as a novel type of actuators, where the actuated joint or link and the actuator are decoupled by means of an elastic element (commonly a spring or an elastic tendon). These actuators help to limit issues related to high impedance, which is undesired in wearable robots [8], and holds promising potential to overcome the limitations of actuators in wearable assistive devices as bioinspired designs are considered [9]. A remarkable feature of such systems is the ability to adjust actuation stiffness according to circumstances, allowing them to provide safe interactions with humans under unknown conditions [10]. Considering this approach, multiple wearable assistive devices have been developed. Regarding exoskeletons, platforms that incorporate elastic actuators by means of Bowden cables attached to each joint can be found in the literature, allowing the exoskeleton to be lightweight, safe, and to provide better interaction with the user [11]. Likewise, tendon-driven exoskeletons have been designed for elderly care, providing safe and compliant actuation for patients [12]. Similarly, pneumatic muscle actuators that exhibit high power actuation and more natural interaction have been incorporated in different exoskeleton designs [2,13,14]. On the other hand, for orthotic devices, similar actuation strategies for specific joints have been explored. Specifically, several Ankle–Foot Orthoses (AFO) have been designed, implementing series-elastic actuators [3,15], integrating artificial pneumatic muscles [16] and, most recently, a novel AFO (i.e., T-Flex) that incorporates elastic tendons has been designed to assist stroke patients that suffer from drop foot [17].

As novel actuation approaches are explored in wearable devices, the design of control strategies to provide accurate and robust behaviors of the system is required [1]. In this regard, to enable the complexity of such behaviors, the sensory system plays a key role, since it allows the robotic device to perceive the environment and identify interaction variables [18]. However, conventional electronic sensors present significant limitations in terms of flexibility, robustness, and sensitivity [19]. Therefore, there is the need to develop novel sensors capable of meeting basic requirements, such as compliance (i.e., avoid restrictions or modifications of the soft device’s properties) and resilience (i.e., avoid failure along repetitive operation cycles) [1]. Moreover, as most of these sensors are meant to be integrated with soft structures, they must exhibit elastic properties that allow them to operate under conditions of high stress and deformation. Hence, such sensors must be composed of materials that typically present properties in the order of <1 MPa for the elastic modulus, and >200% for maximum strain [1].

In the context of wearable soft robotics, most sensors are designed to measure strain and position [20]. Hence, considering the requirements that such sensors must satisfy to operate, a varied number of technologies for the instrumentation of these devices have emerged. Among them, optical-fiber technology has promising potential in soft-robotics applications [19]. Unlike conventional sensors, optical fibers are not affected by electromagnetic interference as they only need a means to transmit light, which make them suitable to operate in environments where conventional sensors have difficulties [21]. In general, there are two main types of optical fibers: silica optical fibers and polymer optical fibers (POFs), which are preferable for sensing applications due to their mechanical properties (e.g., flexibility, impact resistance, and high strain limits) [22]. Among operation principles, there are two main approaches that are commonly implemented, namely, Fiber Bragg Grating (FBG) and power variation. FBG sensors have been broadly implemented for several applications, such as temperature sensing [23], strain [24], structural health monitoring [25], accelerometers [26], and force sensors [27]. However this technology requires high-cost lasers for grating inscription, and interrogation equipment generally has low portability and high cost [28].

Unlike FBG technology, power-variation sensors offer major advantages regarding low-cost equipment, portability, and simplicity for signal processing, which make them suitable for wearable applications [29]. From this approach, different applications have implemented optical sensors, such as bending sensors to track spinal posture [30], strain measurement in soft orthosis [31], or optical systems to monitor respiratory and cardiac parameters [32,33]. However, there are applications where higher deformations and elastic properties are required. Hence, different groups have developed soft stretchable strain-sensitive optical fibers to measure strain in fabrics [34], as well as to measure deformation in prosthetic hands [35]. Similarly, hydrogel-based optical fibers have been developed, reporting high stretching properties (>700% strain) [36]. However, these fibers require a more complex fabrication process. Furthermore, another approach is based on the light-coupling principle [37], and several sensors have also been developed to measure the deformation of soft materials [38], interaction forces with exoskeletons [39], displacement [40], and bending angles [41]. One of the main advantages of this approach is the simplicity of the sensors, and applicability in conditions were high deformations are presented. Therefore, this paper presents the implementation of a POF strain-gauge sensor based on a light-coupling principle (that does not depend on the fiber’s viscoelastic properties), aiming to measure the deformation of elastic tendons under tensile stress implemented in the soft-robotic foot–ankle orthosis T-Flex for foot-drop rehabilitation [17]. A relevant aspect that such tendon-driven orthotic devices must exhibit is their ability to online-measure acting forces on both directions (either from orthosis to joint or from user to device) that are transmitted through the tendons.

The main motivation of this work is to present a lightweight optical strain sensor that is easy to fabricate and install on elastic tendons incorporated in series-elastic actuators. Hence, with the implementation of this sensor, it is expected to increase the human–robot interaction capabilities of the device by perceiving the interaction forces (that, to date, are typically analyzed on the actuator), and allow tendon-driven robotic platforms to have multiple information sources about their interaction and implement more robust control strategies that enable them to identify users’ movement intentions without altering the mechanical properties (stress–strain behavior) of the tendon. The remainder of this paper is organized as follows. Section 2 presents the system requirements and features of the robotic orthosis that motivated the development of the sensor. Section 3 describes the operation principle with the analytical approach, followed by Section 4, which presents the experiment procedure to perform the characterization of the sensor with results and the discussion. Finally, conclusions and recommendations for future work are discussed in Section 5.

## 2. Sensor System Requirements

### 2.1. Human Tendons and Bioinspiration

**Figure 1:**
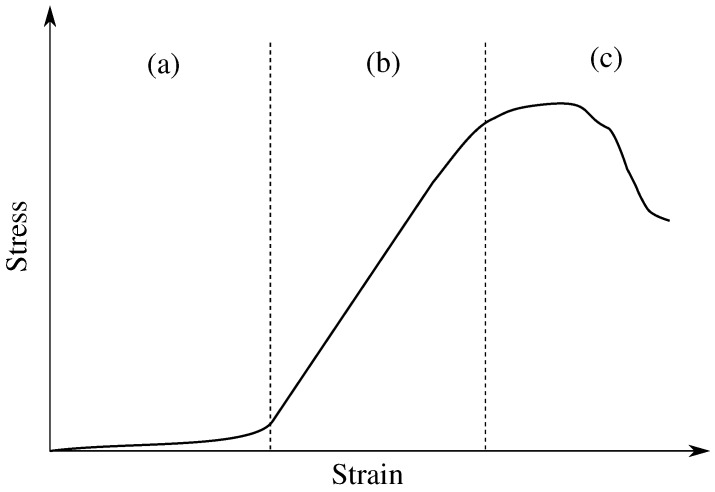
Stress–strain curve behavior of human tendons. **Region** (**a**) elastic behavior of the tendon where high deformation is presented under low stress. **Region** (**b**) linear behavior of tendon where deformation is proportional to applied stress. **Region** (**c**) tendon failure zone.

Human tendons are a type of soft tissue composed of collagen fibers dedicated to transmitting mechanical forces between muscles and bones [42]. The fibril structure of tendons produces anisotropic and nonlinear properties that are reflected in their tensile stress–strain mechanical response [43], generating distinguished regions that exhibit different behaviors that can be analyzed in the load-elongation curve [42]. As illustrated in Figure 1, there are three regions that present different behaviors. Region (a) describes the elastic behavior of the tendon that takes place when the initial load is applied and high deformation is generated. In Region (b) a linear behavior can be observed, where the tendon’s strain increases proportionally to the applied force. Finally, Region (c) illustrates when tendon failure occurs [42,44]. Experiment tests were carried out to determine the material properties, finding average strain ranges between 5% and 16%, failure stresses in the 50–125 Mpa range, and module values ranging from 500 up to 1850 MPa [45].

### 2.2. T-Flex Robotic Orthosis and Actuation System

**Figure 2:**
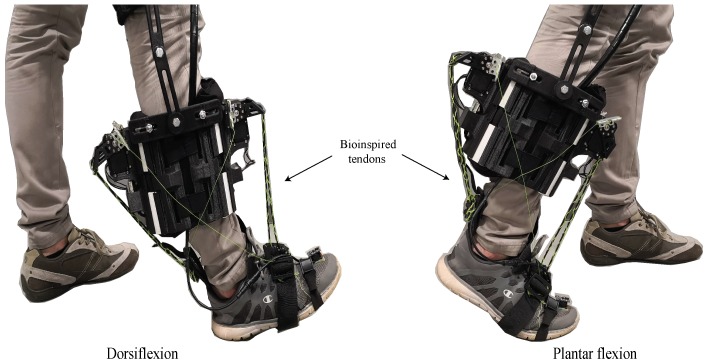
T-Flex: wearable ankle—foot robotic orthoses. Both movements, dorsiflexion and plantar flexion, are produced by means of the elastic tendons attached in series to the servomotors.

T-Flex is a wearable robotic orthosis designed to assist the gait for people with mobility impairments associated with the foot-ankle (e.g., foot drop) (see Figure 2) [17]. This orthosis was designed based on a bidirectional antagonistic variable-stiffness actuator [46] that allows the device to act on the ankle joint through tendon-driven actuators, aiming to reproduce the same human-body principle. The device is composed of two servomotors placed on the posterior and anterior parts of the user’s shank. The motors are attached in series to elastic tendons constructed with the combination of two materials (elastic and rigid filaments) in a configuration that aims to mimic the human tendon’s stiffness and tensile strength. This kind of tendon was first developed in Reference [47], where an actuator control in a printable robot arm, based on the bidirectional antagonistic principle, was proposed. The main goal of this tendon-driven orthosis is to reproduce variable-stiffness actuation profiles where tendons are manipulated in such a way that they can resemble the behavior of two antagonist muscles and allow the variation of the impedance on the ankle at specific walking phases [17].

**Figure 3:**
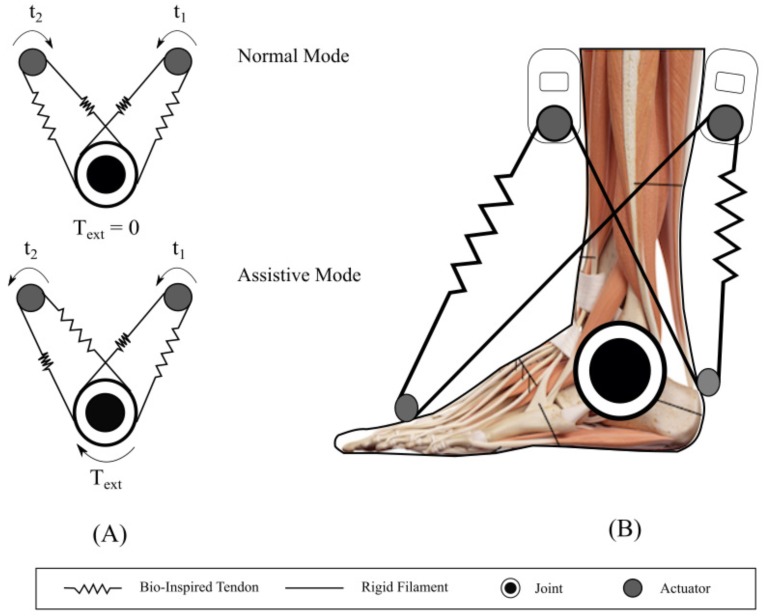
Soft actuation principle for robotic orthosis T-Flex. (**A**) Two operation modes: Normal mode is present when both motors act in opposite directions, generating a null external torque. In this state, there is a constant stiffness on the joint. Assistive mode operates when both motors are actuated in the same direction, generating an external torque. In this state, the actuation system supports the rotation of the joint in any direction. (**B**) Actuation-system configuration for the T-Flex orthosis: Both motors are attached in series to elastic elastic tendons that transmit the axial force to the joint. Depending on operation mode, this configuration allows to stabilize the ankle joint or to support and assist the rotation movement of the ankle.

The actuation principle is illustrated in Figure 3, where two operation modes of the robotic orthosis are described (Figure 3A). The normal mode shows both motors acting in opposite directions, generating a null external torque on the joint. In this mode, the goal is to maintain constant stiffness on the ankle to provide stability. The assistive mode produces external torque on the joint, since both motors act in the same direction, providing assistance when the user performs any movement. Moreover, Figure 3B shows the specific configuration for T-Flex orthosis. Both actuators are attached in series with a tendon that acts as an elastic element, and a rigid filament (fishing rod) that is incorporated to provide more stability during actuation. As described before, when both motors act in the same direction (assistive mode), they produce an external torque that generates the rotation of the ankle joint, whereas in normal mode, the orthosis generates constant stiffness on the joint, helping to stabilize the ankle. As observed in the T-Flex configuration, the actuation system works in parallel with the human tendons; thus, there is more natural actuation and all interaction forces are transmitted trough the elastic tendons. Hence, sensing the tendon’s behavior under axial stress is critical to identify the applied forces in specific moments. Similarly, given the bioinspired design of the orthosis, these tendons must exhibit mechanical properties (axial strain–stress behavior) that are comparable to human tendons.

**Figure 4:**
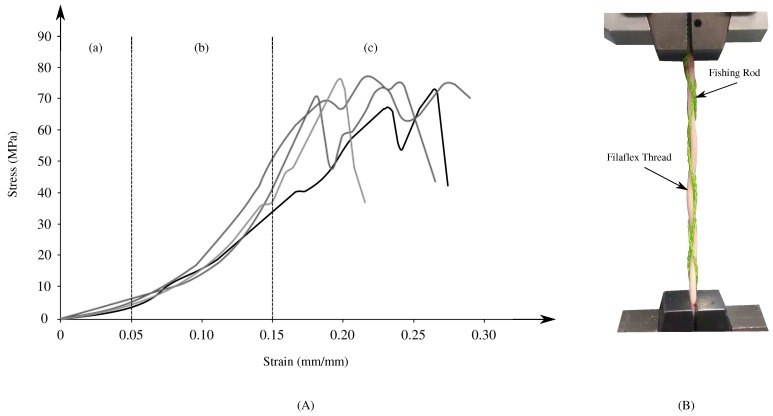
(**A**) Stress–strain curve behavior of elastic tendons integrated in the T-Flex robotic orthosis. Stress–strain curve exhibits similar behavior to the human tendon, where the three characteristic regions (elastic, linear and failure) are observed. An average maximum strain before failure of 15%, average failure stress obtained of 75 MPa, and modules in 150–510 MPa range are found for the combined material; (**B**) material configuration and experiment setup for tensile test.

Considering the human tendon’s mechanical properties, tendon design must resemble similar behavior to be able to actuate and provide adequate stiffness to the foot–ankle joint. Therefore, a combination of two materials (i.e., Filaflex as the elastic component and fishing rod coiled to the Filaflex acting as the rigid component) was designed to generate the stress–strain profile comparable to the human tendon. Results of the experiment tests performed to characterize the material considering different samples are illustrated in Figure 4. As can be observed, similar properties of human tendons are exhibited by the material. The three regions are distinguished, presenting an average maximum strain before failure of 15%. Likewise, the average failure stress obtained was 75 MPa, and moduli in 150–510 MPa range were observed. Thus, with the adequate mechanical behavior of the tendon, a suitable sensing strategy must be analyzed in order to obtain the desired information (i.e., force transmission and tendon deformation) without interfering in orthosis functionality or altering the tendon’s mechanical behavior.

For the experiments, six different samples of tendons with the same configuration were used. As previously described, the tendon is composed by two materials, Recreus Filaflex with a 2.85 mm diameter, and fishing rod Sufix 832 (eight filaments) (Website: https://sufix.fishing/products/832-advanced-superline). Each sample was fabricated with a length of 150 mm and volumetric ratio of 14% (six fishing-rod filaments) with respect to the Filaflex volume. The Filaflex filament counts with a through-hole on each extreme, where the fishing rod is attached. From this point, the six filaments are threaded, wrapping the Filaflex filament (see Figure 4B).

### 2.3. POF Sensor Requirements

Although POFs have great potential for large-strain applications such as infrastructure analysis or shape-changing structures [48,49], it is of relevance to analyze the behavior of the optical fiber in the current application and the performance requirements. In this context, the optical sensor must be able to measure the tendon’s deformation in a range from 0% to 15% and exhibit similar mechanical properties (i.e., Young modulus) as the tendon’s material. Different studies performed experiment tests to define the stress–strain response and the deformation limits of conventional POFs [50]. Results showed a nonlinear response and maximum strain of 15%. However, typical modules are in the range of 3.96–5 GPa [48], while, for the elastic tendon, the stress–strain modulus is in the range of 150–500 MPa (see Table 1).

In this context, as the POF modulus is one order of magnitude greater than the modulus of the tendon, it is not reliable to integrate or embed the fiber with the material (i.e., create a multimaterial configuration Filaflex–Fishing Rod–POF), since it would affect the mechanical properties of the tendon in the axial direction. In preliminary experiments, the PMMA fiber was tested in different scenarios (i.e., removing the plastic coating and performing annealing to increase the flexibility of the fiber). However, these modifications did not meet the mechanical properties expected for this application. For this reason, a configuration that provides adequate performance without altering the mechanical properties of the tendon, such as the light-coupling measurement principle, was selected. This configuration guarantees no alteration of the mechanical properties of the current tendon’s material, since the sensor is decoupled and no axial stress is transmitted through the fiber. The sensor’s operation principle, as well as the mathematical model are presented in the next section.

## 3. Materials and Methods

### 3.1. Sensor Operation Principle

**Figure 5:**
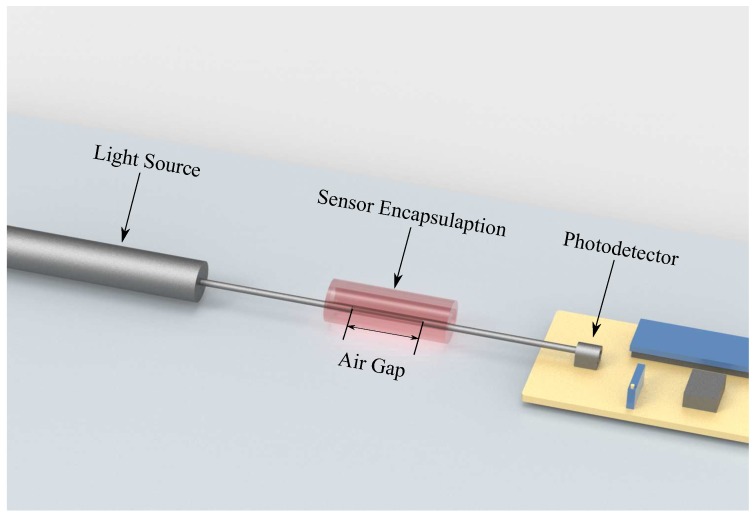
Configuration of optical-fiber strain gauge based on light-coupling principle. Sensor is composed of two fibers, transmitter attached to the light source and receiver attached to the photodetector. In the presence of axial displacement, there is an increment of the air gap that leads to power attenuation.

The sensor’s operation principle is based on light coupling between two POFs, which occurs through axial displacement on the fibers attached to the tendon. In this case, one end of the illuminated POF is connected to a light source, whereas, for the nonilluminated fiber, one end is connected to a photodetector. The other end facet of each POF (illuminated and nonilluminated) is encapsulated as shown in Figure 5 in order to reduce lateral and angular misalignments. Thus, the POFs have a single degree of freedom in the axial direction (see Figure 5). When displacement is applied on the fibers, there is an alignment difference between the two POFs, which leads to power attenuation that is perceived by the photodetector.

### 3.2. Analytical Model

The analytical model considers the POF sensor under different alignment conditions, where POF power variation $\left(\frac{P}{P_{i}}\right)$ (*P* is the coupled power into the nonilluminated fiber and $P_{i}$ is the incident light power) can be estimated for a multimode step index POF, as follows:

(1) $$\left(\frac{P}{P_{i}}\right)=\left[1-\frac{\theta n_{0}}{\pi NA}\right]\left[1-\frac{xNA}{4an_{0}}\right]\left[\frac{\pi }{2}\left(cos^{-1}\left(\frac{1}{2a}\right)-\frac{1}{2a}\sqrt{1-{\left(\frac{1}{2a}\right)}^{2}}\right)\right]$$

where $n_{0}$ is the medium refractive index, and NA and *a* are the POF numerical aperture and core radius, respectively. *l*, θ, and *x* are the lateral, angular, and axial displacements between the fibers, respectively. It is worth to mention that Equation (1) considers uniform power distribution on the POFs [51]. Even though the application of only Equation (1) leads to good approximation when compared with the experiment results under displacements lower than 2 mm [51], the sensor for tendon instrumentation needs to operate in higher displacements conditions (strain >15%), as discussed in the system requirements. Therefore, in high-displacement conditions, it is necessary to consider the attenuation coefficient (μ) of the medium (PMMA POF and air) as well as the path travelled by the light, which is the axial displacement between POFs and the POF length in this case. Hence, power attenuation for higher displacements ${\left(\frac{P}{P_{i}}\right)}_{T}$ can be estimated using Equation (2) [52].

(2) $${\left(\frac{P}{P_{i}}\right)}_{T}=\left(\frac{P}{P_{i}}\right)exp\left(-\mu x\right)$$

Sensor response can be estimated by combining Equations (1) and (2). Hence, to validate this analytical model, an experiment protocol was established to obtain data that can be compared with the proposed model. This protocol is described below.

### 3.3. Experiment Protocol

**Figure 6:**
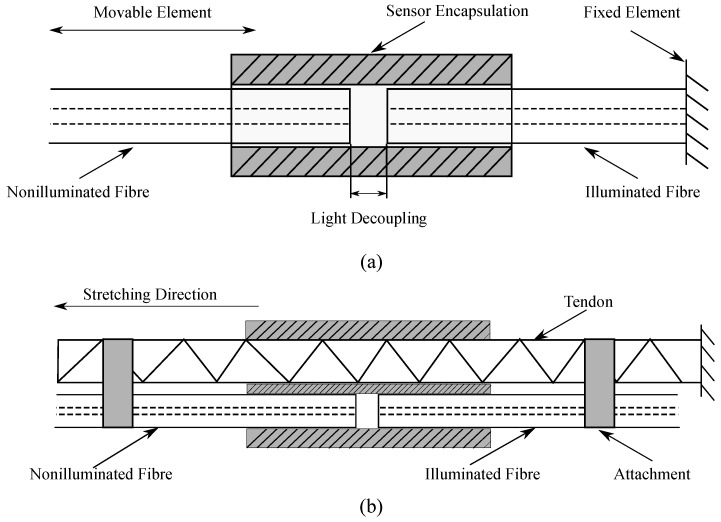
Experiment setup to validate sensor response. (**a**) Two fibers (illuminated and nonilluminated fiber): illuminated fiber is attached to the light source with a fixed element, and nonilluminated fiber is attached to the photodetector with a movable element that allows light decoupling. In order to guarantee only axial displacement, a 3D-printed sensor encapsulation was incorporated to avoid axial misalignment; (**b**) incorporation of the tendon where both fibers are attached by means of a polymer. Tendon is fixed on one extreme, and the other extreme is stretched.

The experiment protocol was divided in two parts. The first setup, designed to validate the proposed analytical model, consisted of two POFs, namely, transmitter and receiver fibers (PMMA with 982 μm core, 10 μm cladding width, and 0.46 numeric aperture ) [49]. The transmitter fiber is attached to a fixed element where the light source is connected, and the receiver fiber is attached to a movable element connected to the photodetector (see Figure 6). Once the sensor response was validated based on the analytical model, the second experiment setup was conducted incorporating the elastic tendon. In this case, both fibers (illuminated and nonilluminated fiber) are attached to both extremes of the tendon (using melted 4 mm polymorph thermoplastic polycaprolactone moldable plastic pellet to glue the fiber to the tendon) and, similar to the first setup (see Figure 6), the extreme of the tendon that was attached to the transmitter fiber was fixed, while the other extreme with the receiver fiber was attached to the mobile element. With this configuration, it was expected that the sensor would track the tendon’s stretching and recovery behavior.

The light source incorporated in the experiments is a laser Phywe 41736-3E, wavelength 650 nm, and optical output power of 3 mW (power consumption 50 mA at 2.2 V). The photodetector was implemented by means of a transimpedance circuit, connected to a microcontroller for signal acquisition with a 16 bit resolution ADC (76.29 μV/bit and sample rate of 3.4 Hz), both circuits being portable and viable to be powered with battery. A 3D-printed sensor encapsulation was designed to avoid significant axial misalignment along the operation range. The experiments were carried out by progressively increasing the axial displacement of the movable element driven by a micrometer screw. This procedure was performed in steps of 1 mm, starting from zero displacement up to a maximum displacement of 15 mm, and returning to the initial position in the same way. For both experiment setups, 3 repetitions were carried out. Results are presented in the next section.

## 4. Results and Discussion

**Figure 7:**
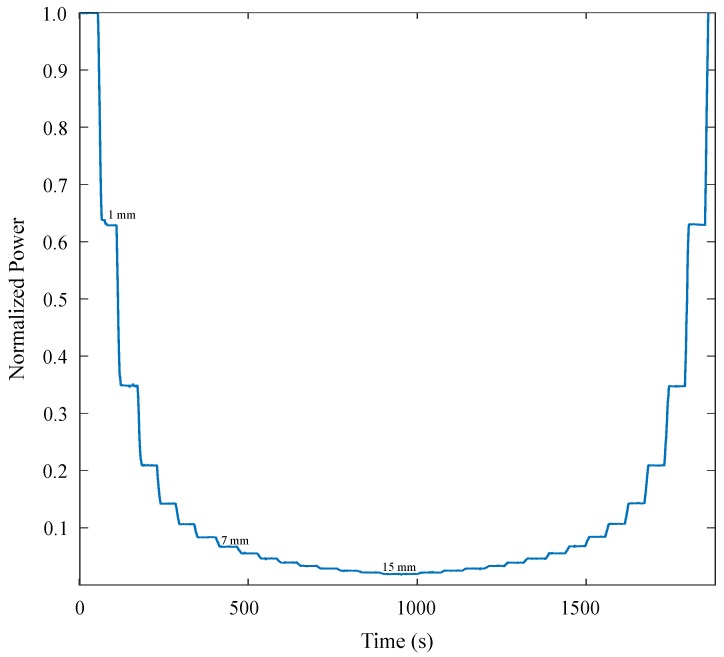
Optical strain-gauge dynamic response to axial displacement. Experiment was carried out by increasing axial displacement in steps of 1 mm and remaining in each position for a considerable period of time to analyze signal stability at each point, starting from zero displacement (maximum light coupling), until a maximum displacement of 15 mm, where almost the maximum light decoupling (reflected in the transmitted power) was presented. Once the sensor reached axial displacement of 15 mm, it was returned to the original position following the same procedure.

This section first presents the results of sensor validation according to the analytical model. As was described in the previous section, axial displacement was progressively increased in steps of 1 mm. Results of the sensor response are illustrated in Figure 7, where the normalized power is plotted in a function of time. As illustrated, the experiment consisted of performing displacements of 1 mm and remaining in that position for a considerable amount of time to analyze signal stability. Displacements started from zero displacement, where the maximum light coupling is reached, until 15 mm, and then returned to the zero position in the same way. It can be observed that, for the first axial displacements, variation of the coupled power is high while, for larger displacements, power variation decreases, showing exponential behavior.

**Figure 8:**
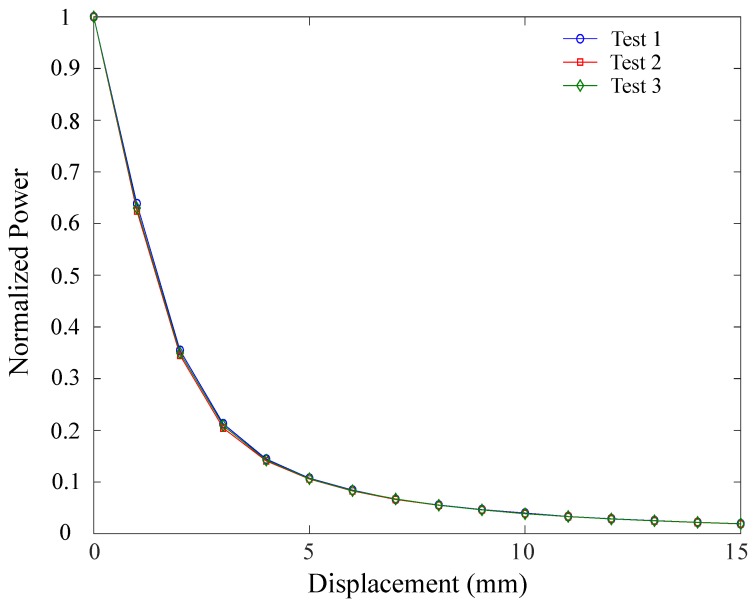
Optical strain-gauge response. Normalized power attenuation in function of axial displacement (mm) is illustrated for three different tests.

The aforementioned procedure was performed for three tests. Hence, the normalized power response as a function of sensor axial displacement for the three experiments is shown in Figure 8. This plot reflects the high repeatability of the experiments, where low variation (correlation coefficient, R = 0.97) is presented between tests. Similarly, hysteresis was numerically quantified by means of the root mean square(rmse), where difference between the stretching (increasing axial displacement) and relaxing (decreasing axial displacement) phases was estimated. For these experiments, rmse=0.012 was found, indicating only a 1.2% difference between both phases. Thus, the sensor presents a good repeatability index and low hysteresis.

**Figure 9:**
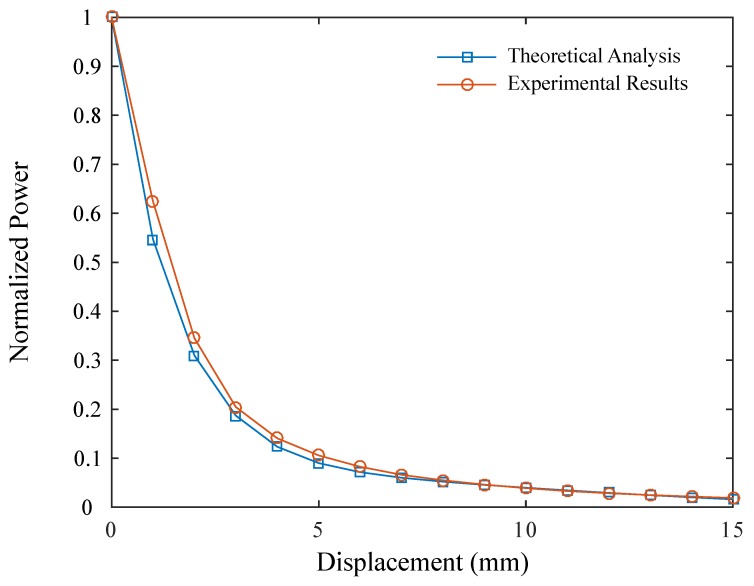
Experiment results for sensor-model validation. theoretical analysis (blue) compared with experiment data (red).

Furthermore, in order to verify the analytical model, the sensor response was simulated for axial displacement from 0 to 15 mm, where the estimated response was compared with the obtained experiment data in the same displacement range. Table 2 shows the constants used on the analytical analysis of the sensor response. However, the attenuation coefficient of the air was unknown. For this reason, exponential regression was made on the experiment results, and an attenuation value of 50 dB/km was obtained. Figure 9 shows the obtained results using the proposed model and those measured on the experiment evaluation. Comparing the results obtained using the theoretical and experiment approaches, correlation coefficient R=0.998 was obtained, which is regarded as high correlation between the two results. Thus, the proposed displacement sensor operates as predicted by the model and could be applied on measuring displacements up to 15 mm, which is within the strain range (ratio between displacement and initial length) of the humanlike tendon.

**Figure 10:**
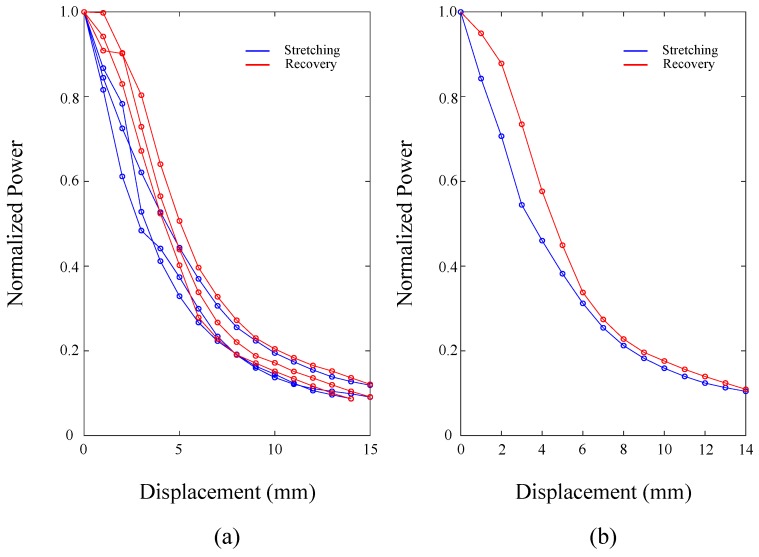
Results of experiment tests carried out with the elastic tendon. (**a**) Curves of normalized power vs. displacement for three tests performed with different tendons of the same characteristics. Blue lines, stretching phase; red, recovery phase. (**b**) Average behavior of the three experiments for both phases; stretching (blue) and recovery (red). Hysteresis is presented.

Once the sensor analytic model was validated with the experiment data, the second experiment setup was carried out, aiming to obtain the deformation response of the tendon. Hence, the same protocol applied for sensor validation was conducted. Results are presented in Figure 10. Curves (normalized power vs. displacement) of three tests performed with equally constructed tendons are illustrated in Figure 10a. Blue lines represent the stretching phase, whereas red lines represent the recovery phase of the tendons after stretching. Unlike the obtained data for the characterization of the sensor presented before, where no significant hysteresis was present, it could be observed that, for all tests, there was considerable difference between tendon stretching and recovery. As the correlation between the three tests was high (R > 0.97), the mean curve was analyzed. Figure 10b illustrates the mean curve of the three experiments considering the stretching and recovery phases. As observed in the figure, there was notable difference between stretching and recovery. Hysteresis, numerically estimated through the rmse, was found to be rmse=0.204, which is significantly high when compared to hysteresis presented only for the sensor (20.4%, sensor tendon vs. 1.2%, sensor). According to these results, the difference between the experiments can be attributed to existing variability in the construction of each tendon. Since material configuration can present differences, the response between different samples is also prone to variation. Similarly, the material behaves differently between phases. Under tensile stress, both materials (fishing rod and Filaflex) react to the applied force. However, in the relaxation phase, the elastic material returns to its original condition, while the fishing rod has no significant participation. Thus, hysteresis found in the tendon could be attributed to this condition.

**Figure 11:**
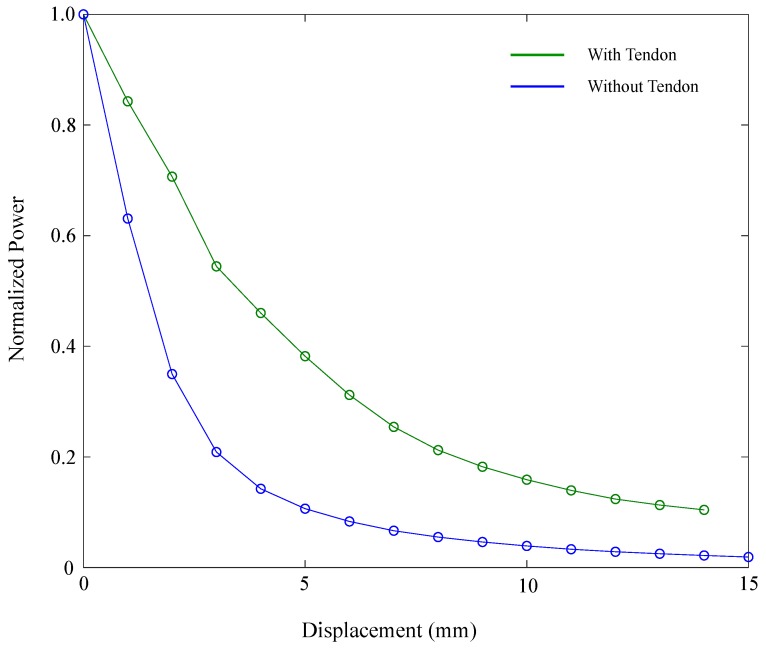
Comparison between isolated sensor response (blue) and response of sensor attached to the tendon (green).

As evidenced in Figure 10, when the sensor is attached to the elastic tendon, its response presents significant differences compared with the isolated sensor. At first, unlike the first experiments without the tendon, the results of the three tests present variations between themselves, and considerable hysteresis was observed. Additionally, the power-attenuation response also changed. Figure 11 illustrates both responses for the stretching phase, where the isolated sensor’s response is plotted in blue, and the response of the sensor attached to the tendon is plotted in green. The figure shows that the green plot was shifted upward, which indicates that lower attenuation is obtained for the same displacement of the element. This behavior can be explained due to the structure of the sensor and the way it is attached to the tendon. As shown in Figure 6b, both fibers are glued to the tendon; hence, both elements present displacement. Unlike the original setup ( Figure 6a), where the illuminated fiber is fixed and the relative displacement is the same as the displacement of the movable element, for the tendon measurement, relative displacement is different with respect to the total elongation of the tendon. Thus, Equation (2) must be modified with $\delta_{x}$ that acts as an offset factor (see Equation (3)).

(3) $${\left(\frac{P}{P_{i}}\right)}_{T}=\left(\frac{P}{P_{i}}\right)exp\left(-\mu \delta_{x}x\right)$$

**Figure 12:**
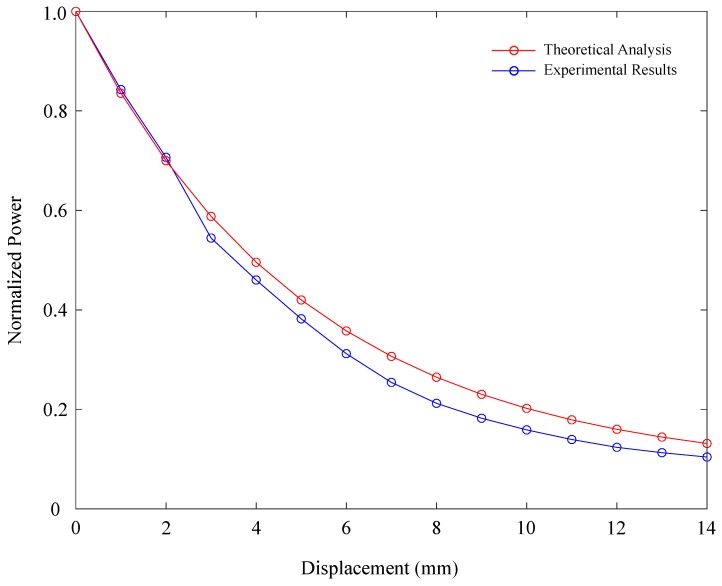
Analytical model adjusted (red line) to the sensor response attached to the elastic tendon (blue line), considering relative displacement between receiver and transmitter fiber.

The factor indicates $\delta_{x}$ that, for total tendon elongation *x*, the relative displacement between receiver and transmitter is a factor of this displacement. In other words, the real attenuation of the sensor presented indicates a light-decoupling distance of $\delta_{x}x$ that is smaller than the total elongation of the tendon. For the current configuration, it was found that a factor $\delta_{x}=0.3$ adapts well to the experiment response, indicating that, for each 1 mm that the tendon was elongated, light-decoupling distance would be 0.3 mm. Figure 12. illustrates the adjusted model response vs. the experiment response obtained with the tendon. As shown in the plot, it can be observed that the model adjusts better to the sensor’s behavior with a correlation coefficient (R) of 0.9625.

Highly compliant and stretchable optical waveguides were fabricated to operate as bending, elongation, and pressure sensors, presenting mechanical properties that reach 100% strain, with elastic moduli ranging from 300 to 400 kPa, which make them suitable for the proposed application [35]. Similarly, hydrogel optical fibers have been reported, reaching up to 700% axial strain with elastic moduli in the range of 80–230 kPa, tested over multiple cycles [36]. An advantage of the proposed sensor configuration is its simplicity and replicability, as it only requires commercially available optical fibers and no additional fabrication process is required. Hence, for the current application, the sensor response was satisfactory, showing promising potential in future applications. Nevertheless, depending on the application, it is important to consider limitations that could appear. First, regarding 3D-printed sensor encapsulation, as the mechanical measurement principle is based on the displacement of the receiver fiber, friction between fiber and encapsulation must be considered depending on the application, since the sensor’s dynamic response could be influenced by this variable. Second, limitations regarding the fiber coupling must be considered. As the edge surface has imperfections at the microscale, the initial condition (zero axial displacement) could be affected, generating difficulties in having a reference value. Finally, depending on the tendon configuration, the $\delta_{x}$ factor must be estimated, since tendon displacement can present differences. Similarly, the attachment to the tendon can present some difficulties depending on the surface conditions of the tendons. This issue can lead to inaccuracies in several dynamic cycles, since the sensor could be detached from the tendon. In this context, further work is addressed to identify the sensor response and behavior in applications that propose more challenging conditions.

## 5. Conclusions and Future Work

This article presented the development and validation of an optical-fiber strain-gauge sensor based on the light-coupling principle for axial strain measurement in elastic tendons incorporated in wearable assistive robots. First, the analytical model was derived based on operation conditions and system requirements. Similarly, experiment data were obtained by means of three tests that provided information to validate the analytical model. From this validation, we found an exponential response for large axial displacement, with a correlation of 0.998 between the analytical model and experiment data, and a maximum measurement range of 15 mm. Furthermore, experiment tests were also carried out to evaluate the sensor in real applications. Hence, the sensor was attached to the tendon, aiming to determine tendon deformation under tensile stress. Results showed that the sensor’s response differed from the obtained results for the isolated sensor, as the power-attenuation curve was shifted upward from the original. Therefore, an adjusted model was proposed, finding a correlation coefficient of 0.9625. From these observations, there is potential for this sensor to determine dynamic tendon behaviors, allowing for a better understanding of the material and better control strategies.

Future work is proposed that would aim at evaluating sensor performance in different potential applications. Hence, as further validation, an experiment is proposed incorporating the sensor in wearable robotic foot–ankle orthosis T-Flex [53]. With this experiment, it is expected to evaluate the response time of the sensor and determine how physical and mechanical limitations can be improved, as well as the dynamic behavior of the elastic tendon. Furthermore, incorporation of this sensor in soft-actuated social robotic platforms to identify human–robot-interaction variables will be considered in future applications. Finally, considering that one of the main limitations of the sensor is the attachment to the elastic element, future work will explore embedding techniques that allow to improve the sensor’s mechanical performance.

## Figures and Tables

**Table 1:** Stress–strain modulus for specific strain ranges of Polymer Optical Fiber (POF) and the elastic tendon.

POF		Elastic Tendon	
Strain Range (%)	Modulus (GPa)	Strain Range (%)	Modulus (MPa)
0–1.0	3.96	0–10	150
1.0–3.0	4.34	10–15	500
3.0–5.0	5.04	>15	500–600

**Table 2:** Analytical model parameters.

Symbol	Parameter	Value
*a*	Fiber radius	0.5 mm
$n_{0}$	Medium refractive index	1.0003
*l*	Lateral misalignment	0.5 mm
θ	Angular misalignment	0 rad
NA	Fiber numerical aperture	0.47
$\mu_{1}$	POF attenuation coefficient	12 dB/km

## Abbreviations

The following abbreviations are used in this manuscript:

POF	Polymer Optical Fiber
AFO	Ankle–Foot Orthoses

## References

[1] Polygerinos P., Correll N., Morin S.A., Mosadegh B., Onal C.D., Petersen K., Cianchetti M., Tolley M.T., Shepherd R.F. (2017). Soft Robotics: Review of Fluid-Driven Intrinsically Soft Devices; Manufacturing, Sensing, Control, and Applications in Human-Robot Interaction. Adv. Eng. Mater..

[2] Huo W., Mohammed S., Moreno J.C., Amirat Y. (2016). Lower Limb Wearable Robots for Assistance and Rehabilitation: A State of the Art. IEEE Syst. J..

[3] Viteckova S., Kutilek P., Jirina M. (2013). Wearable lower limb robotics: A review. Biocybern. Biomed. Eng..

[4] Mohammed S., Amirat Y., Rifai H. (2012). Lower-Limb Movement Assistance through Wearable Robots: State of the Art and Challenges. Adv. Robot..

[5] Moreno J., Asin G., Pons J., Cuypers H., Vanderborght B., Lefeber D., Ceseracciu E., Reggiani M., Thorsteinsson F., Del-Ama A. (2014). Symbiotic Wearable Robotic Exoskeletons: The Concept of the BioMot Project J.C..

[6] Veale A.J., Xie S.Q. (2016). Towards compliant and wearable robotic orthoses: A review of current and emerging actuator technologies. Med. Eng. Phys..

[7] Albu-Schaffer A., Fischer M., Schreiber G., Schoeppe F., Hirzinger G. Soft robotics: What Cartesian stiffness can obtain with passively compliant, uncoupled joints?. Proceedings of the 2004 IEEE/RSJ International Conference on Intelligent Robots and Systems (IROS).

[8] Zinn M., Khatib O., Roth B., Salisbury J.K. (2003). A New Actuation Approach for Human Friendly Robot Design. Exp. Robot. VIII.

[9] Koganezawa K., Ban S. Stiffness control of antagonistically driven redundant D.O.F. manipulator. Proceedings of the IEEE/RSJ International Conference on Intelligent Robots and Systems.

[10] Manti B.M., Cacucciolo V., Cianchetti M. (2016). Stiffening in Soft Robotics. IEEE Robot. Autom. Mag..

[11] Veneman J.F., Ekkelenkamp R., Kruidhof R., Van Der Helm F.C., Van Der Kooij H. Design of a series elastic- and bowdencable-based actuation system for use as torque-actuator in exoskeleton-type training. Proceedings of the 9th International Conference on Rehabilitation Robotics.

[12] Kong K., Jeon D. (2006). Design and control of an exoskeleton for the elderly and patients. IEEE/ASME Trans. Mechatron..

[13] Tsagarakis N., Caldwell D.G. (2003). Development and Control of a ‘Soft-Actuated’ Exoskeleton for Use in Physiotherapy and Training. Auton. Robot..

[14] Costz N., Kousidou S., Caldwell D.G., Tsagarakits N.G., Sarakoglou I. (2007). “Soft” Exoskeletons for Upper and Lower Body Rehabilitation—Design, Control and Testing. Int. J. Humanoid Robot..

[15] Kim J., Hwang S., Sohn R., Lee Y., Kim Y. (2011). Development of an active ankle foot orthosis to prevent foot drop and toe drag in hemiplegic patients: A preliminary study. Appl. Bionics Biomech..

[16] Cain S.M., Gordon K.E., Ferris D.P. (2007). Locomotor adaptation to a powered ankle-foot orthosis depends on control method. J. Neuroeng. Rehabil..

[17] Manchola M., Serrano D., Daniel G., Ballen F., Casas D., Munera M. Wearable Robotics: Challenges and Trends. Proceedings of the 2nd International Symposium on Wearable Robotics—WeRob2016.

[18] Rossiter J., Hauser H. (2016). Soft Robotics—The Next Industrial Revolution? [Industrial Activities]. IEEE Robot. Autom. Mag..

[19] Leal-Junior A., Casas J., Marques C., Pontes M., Frizera A., Leal-Junior A., Casas J., Marques C., Pontes M.J., Frizera A. (2018). Application of Additive Layer Manufacturing Technique on the Development of High Sensitive Fiber Bragg Grating Temperature Sensors. Sensors.

[20] Goldfield E.C., Park Y.L., Chen B.R., Hsu W.H., Young D., Wehner M., Kelty-Stephen D.G., Stirling L., Weinberg M., Newman D. (2012). Bio-Inspired Design of Soft Robotic Assistive Devices: The Interface of Physics, Biology, and Behavior. Ecol. Psychol..

[21] Pinet É. (2009). Fabry-Pérot Fiber-Optic Sensors for Physical Parameters Measurement in Challenging Conditions. J. Sens..

[22] Leal-Junior A.G., Frizera A., Vargas-Valencia L., Dos Santos W.M., Bo A.P., Siqueira A.A., Pontes M.J. (2018). Polymer Optical Fiber Sensors in Wearable Devices: Toward Novel Instrumentation Approaches for Gait Assistance Devices. IEEE Sens. J..

[23] James S.W., Tatam R.P. (2003). Optical fibre long-period grating sensors: Characteristics and application. Meas. Sci. Technol..

[24] Patrick H., Williams G., Kersey A., Pedrazzani J., Vengsarkar A. (1996). Hybrid fiber Bragg grating/long period fiber grating sensor for strain/temperature discrimination. IEEE Photonics Technol. Lett..

[25] Chan T., Yu L., Tam H., Ni Y., Liu S., Chung W., Cheng L. (2006). Fiber Bragg grating sensors for structural health monitoring of Tsing Ma bridge: Background and experimental observation. Eng. Struct..

[26] Guo T., Liu F., Guan B.O., Albert J. (2016). Tilted fiber grating mechanical and biochemical sensors. Opt. Laser Technol..

[27] Shao L.Y., Albert J. (2011). Lateral force sensor based on a core-offset tilted fiber Bragg grating. Opt. Commun..

[28] James S., Dockney M., Tatam R. (1996). Simultaneous independent temperature and strain measurement using in-fibre Bragg grating sensors. Electron. Lett..

[29] Bilro L., Alberto N., Pinto J.L., Nogueira R. (2012). Optical sensors based on plastic fibers. Sensors (Switzerland).

[30] Dunne L.E., Walsh P., Smyth B., Caulfield B. Design and evaluation of a wearable optical sensor for monitoring seated spinal posture. Proceedings of the 2006 10th IEEE International Symposium on Wearable Computers.

[31] Zhao B.H., Jalving J., Huang R., Knepper R., Ruina A., Shepherd R. (2016). A Helping Hand: Soft Orthosis with Integrated Optical Strain Sensors and EMG Control. IEEE Robot. Autom. Mag..

[32] Jae Yoo W., Won Jang K., Ki Seo J., Yeon Heo J., Soo Moon J., Park J.Y., Lee B. (2010). Development of Respiration Sensors Using Plastic Optical Fiber for Respiratory Monitoring Inside MRI System. J. Opt. Soc. Korea.

[33] Grillet A., Kinet D., Witt J., Schukar M., Krebber K., Pirotte F., Depré A. (2008). Optical Fiber Sensors Embedded Into Medical Textiles for Healthcare Monitoring. IEEE Sens. J..

[34] Harnett C.K., Zhao H., Shepherd R.F. (2017). Stretchable Optical Fibers: Threads for Strain-Sensitive Textiles. Adv. Mater. Technol..

[35] Optoelectronically Innervated Soft Prosthetic Hand via Stretchable Optical Waveguides. https://pdfs.semanticscholar.org/70c4/4a842f20ff3c45d74d6e2e6653bcc40ef388.pdf.

[36] Guo J., Liu X., Jiang N., Yetisen A.K., Yuk H., Yang C., Khademhosseini A., Zhao X., Yun S.H. (2016). Highly Stretchable, Strain Sensing Hydrogel Optical Fibers. Adv. Mater..

[37] Taffoni F., Formica D., Saccomandi P., Pino G., Schena E., Taffoni F., Formica D., Saccomandi P., Pino G.D., Schena E. (2013). Optical Fiber-Based MR-Compatible Sensors for Medical Applications: An Overview. Sensors.

[38] To C., Hellebrekers T., Jung J., Yoon S.J., Park Y.L. (2018). A Soft Optical Waveguide Coupled With Fiber Optics for Dynamic Pressure and Strain Sensing. IEEE Robot. Autom. Lett..

[39] Leal-Junior A.G., Frizera A., Marques C., Sánchez M.R., Botelho T.R., Segatto M.V., Pontes M.J. (2018). Polymer optical fiber strain gauge for human-robot interaction forces assessment on an active knee orthosis. Opt. Fiber Technol..

[40] Vallan A., Casalicchio M.L., Olivero M., Perrone G. (2012). Assessment of a Dual-Wavelength Compensation Technique for Displacement Sensors Using Plastic Optical Fibers. IEEE Trans. Instrum. Meas..

[41] Zawawi M.A., O’Keeffe S., Lewis E. (2013). Plastic optical fibre sensor for spine bending monitoring with power fluctuation compensation. Sensors (Switzerland).

[42] Beach Z.M., Gittings D.J., Soslowsky L.J. (2018). Muscle and Tendon Injuries. Med. Sci. Sports Exerc..

[43] Svensson R.B., Hassenkam T., Hansen P., Peter Magnusson S. (2010). Viscoelastic behavior of discrete human collagen fibrils. J. Mech. Behav. Biomed. Mater..

[44] Atkinson T.S., Ewers B.J., Haut R.C. (1999). The tensile and stress relaxation responses of human patellar tendon varies with specimen cross-sectional area. J. Biomech..

[45] Wren T.A.L., Yerby S.A., Beaupr G.S., Carter D.R., Beaupré G.S. (2001). Mechanical properties of the human achilles tendon. Clin. Biomech..

[46] Petit F., Chalon M., Friedl W., Grebenstein M., Albu-sch A. Bidirectional Antagonistic Variable Stiffness Actuation: Analysis, Design & Implementation. Proceedings of the 2010 IEEE International Conference on Robotics and Automation.

[47] Bonsignorio F., Cangelosi A. Co-exploring Actuator Antagonism and Bio-inspired Control in a Printable Robot Arm. Proceedings of the 14th International Conference on Simulation of Adaptive Behavior, SAB 2016.

[48] Kiesel S., Peters K., Hassan T., Kowalsky M. (2007). Behaviour of intrinsic polymer optical fibre sensor for large-strain applications. Meas. Sci. Technol..

[49] Krebber K., Lenke P., Liehr S., Witt J., Schukar M. (2008). Smart technical textiles with integrated POF sensors. Proc. SPIE 6933 Smart Sens. Phenom. Technol. Netw. Syst..

[50] Welker D.J., Johns W.E., Jiang C., Ding J.L., Kuzyk M.G. (2002). Fabrication and mechanical behavior of dye-doped polymer optical fiber. J. Appl. Phys..

[51] Antunes P.F.C., Varum H., Andre P.S. (2013). Intensity-encoded polymer optical fiber accelerometer. IEEE Sens. J..

[52] Ziemann Q., Krauser J., Zamzow P.E. (2008). POF Handbook—Optical Short Range Transmission Systems.

[53] Sánchez-Manchola M., Gómez-Vargas D., Casas-Bocanegra D., Múnera M., Cifuentes C.A. Development of a Robotic Lower-Limb Exoskeleton for Gait Rehabilitation: AGoRA Exoskeleton. Proceedings of the 2018 IEEE ANDESCON.

